# Functional Impairment Related to ADHD From Preschool to School Age

**DOI:** 10.1177/10870547241301179

**Published:** 2024-12-03

**Authors:** Kristin Romvig Overgaard, Beate Oerbeck, Svein Friis, Are Hugo Pripp, Heidi Aase, Christine Baalsrud Ingeborgrud, Guido Biele

**Affiliations:** 1 Oslo University Hospital, Norway; 2 University of Oslo, Norway; 3 Norwegian Institute of Public Health, Oslo, Norway

**Keywords:** ADHD, comorbidity, impairment, longitudinal

## Abstract

**Objective:**

Children with ADHD often experience functional impairments across various aspects of daily life. This study addresses the dearth of longitudinal research on functional impairment trajectories from preschool to school age in children with symptoms of ADHD and comorbid disorders.

**Methods:**

We investigated the extent to which functional impairments were associated with ADHD symptoms, along with behavioral and anxiety symptoms, from age 3.5 to 8 years. Utilizing parent- and teacher-reported data, we analyzed associations between global impairment, and dimension scores (e.g., family; child quality of life (QoL); learning; play/leisure activities; and friends) and symptoms at ages 3.5 and 8 years (*n* = 783).

**Results:**

The mean parent global impairment score increased from 0.31 (standard deviation (*SD*) = 0.40) to 0.83 (*SD* = 0.63) from 3.5 to 8 years, while the teacher impairment scores slightly decreased. Specific parent impairment dimension scores, particularly QoL, learning, and friends, significantly increased. Preschool ADHD and comorbid behavioral symptoms reported by parents weakly predicted impairment at 8 years. By age 8 years, impairment and symptoms exhibited moderate to strong correlations for all impairment dimensions. Parents reported greater child impairment during school age across settings compared to preschool, while teachers’ impairment profiles remained consistent across ages.

**Conclusions:**

These findings suggest that parents perceive impairment as more pronounced at age 8 years and more strongly associated with symptoms of both ADHD and comorbid disorders than at age 3.5 years. Notably, for teachers, a robust correlation between inattention symptoms and learning impairment was observed, with substantially higher impairment scores reported for boys compared to girls.

Children with ADHD often experience functional impairments across various aspects of daily life (Spencer et al., 2007). Some studies suggest that symptoms of hyperactivity–impulsivity (HI) and inattention (IA) may have distinct influences on functioning. Hyperactivity is linked to peer rejection and relational aggression, while inattention is associated with academic challenges, social withdrawal, and poor adaptive functioning (Lahey et al., 1998; Willcutt et al., 2011, 2012). These insights primarily originate from cross-sectional studies involving children beyond the preschool stage, posing challenges in understanding developmental trajectories and generalizing findings to younger children. A review emphasized that children who show a decline in ADHD symptoms from preschool onwards may still encounter impairments in school age (O’Neill et al., 2017). Limited longitudinal research exists on early childhood, with some studies suggesting a pattern of a notable decrease in HI symptoms and stable or increasing IA symptoms over time (Curchack-Lichtin et al., 2014; Overgaard et al., 2023; Riddle et al., 2013). However, these studies offer minimal insights into the longitudinal development of impairment or the predictive power of early ADHD symptoms and common comorbidities, like behavioral and anxiety symptoms (Gnanavel et al., 2019) on subsequent impairment. To address this gap, we conducted a literature search using the terms “ADHD and impairment and preschool” and “longitudinal and/or comorbidity” (until March 15th, 2024). The search yielded only six studies that specifically addressed preschool data, and only two included preschool impairment (Lahey et al., 2016; Leopold et al., 2019). Both studies started assessments after the age of 4 years and found stable overall impairment from preschool to school age.

The first of these two studies identified in the literature search, conducted by Leopold et al. (*n* = 489, twins, first assessment at age 4.9 years), found that HI and IA symptoms increased the risk of concurrent and future overall, social, and recreational impairment, with IA being significantly associated with academic impairment (Leopold et al., 2019). However, that study was constrained by its reliance on parent reports and focused solely on twins. The authors emphasized the need for future longitudinal investigations to compare ADHD with and without comorbidity to elucidate distinct symptom trajectories and levels of impairment. The second identified longitudinal study, by Lahey et al. (2016), observed that children diagnosed with ADHD at age 4 to 6 years (*n* = 125) exhibited more symptoms and greater global impairment (measured by the Children’s Global Assessment Scale (CGAS)) and engaged in various risky behaviors (e.g., driving and crime) through adolescence compared to controls (*n* = 130). That study also indicated that preschool IA and comorbid behavioral- and anxiety symptoms predicted poorer global functioning, though it did not investigate specific impairment domains (Lahey et al., 2016). A population study, with initial assessments at age 3.6 years, followed the children until 17.7 years (*n* = 473) but did not include data on preschool impairment. Nevertheless, it reported that early ADHD diagnoses (at ages 3 or 6 years) significantly predicted worse academic functioning and poorer peer functioning in adolescents, even though the participants demonstrated minimal overall global impairment (mean CGAS = 80.58; Finsaas et al., 2020). These findings ought to be considered preliminary due to the low number of longitudinal studies beginning during preschool age. However, they are in line with two reviews from two decades ago, which concluded that preschoolers with ADHD exhibit psychosocial impairments in relationships and functioning at home and in preschool, similar to findings in studies of older children (Egger & Angold, 2006; Egger et al., 2006). In a previous cross-sectional study at age 3.5 years from our group, it was found that impairment within the family domain was particularly pronounced, whereas other dimensions were less impacted (Bendiksen et al., 2014). In our longitudinal follow-up study to age 5 years, classifying children with ADHD, yielded similar predictive values for impairment as for ADHD symptoms at age 3.5 years (Overgaard et al., 2022). This suggests that early impairment may indeed be important, yet specific impairment dimensions were not investigated.

As for sex differences in impairment, we identified one review article. It suggested that, in non-referred samples, boys exhibited more impairment than girls, whereas similar levels of impairment between boys and girls were observed in clinical samples (Rapee et al., 2012). The authors emphasized the difficulty of interpreting these findings, given variations with age, informant, and the domain of impairment. A more recent population study found that the relationship between ADHD symptom severity levels and impairment was not altered when controlling for sex (Arildskov et al., 2022). Consequently, it remains an open question whether parents and teachers systematically differ in their reports of impairment between boys and girls.

In summary, there is a dearth of longitudinal studies on ADHD and the trajectories of impairment from preschool to school age. A significant knowledge gap exists concerning the developmental trajectories of HI and IA and their relationship to concurrent and future impairment, including information about comorbid symptoms. Based on the available literature, we have formulated the following hypotheses: (1) Scores of several impairment dimensions, as reported by parents and teachers, will increase from 3 to 8 years; (2) Parent-reported scores of HI, IA, and comorbid anxiety and behavioral symptom at baseline will moderately predict impairment at 8 years; (3) For both parents and teachers, scores of HI, IA, and comorbid symptoms at 8 years will be significantly associated with impairment at 8 years; (4) There will be no clear sex difference in parent-reported impairment scores; (5) Teachers will rate girls with lower levels of impairment than boys.

## Methods

### Participants

The Norwegian Mother, Father, and Child Cohort Study (MoBa) is an ongoing population-based cohort study conducted by the Norwegian Institute of Public Health. From 1999 to 2008, pregnant Norwegian-speaking women (predominantly white Caucasians) undergoing their first ultrasound were enrolled from all over Norway (*n* ~ 114,500 children; 41% participation rate; Magnus et al., 2016). The mean household income was approximately 40,000 euros, matching the population average (Overgaard et al., 2014). Nested within MoBa is an ADHD sub study, which oversampled for hyperactive children based on ADHD symptoms from a questionnaire used at child age 36 months in the cohort study, a procedure previously described in detail (Overgaard et al., 2018, 2019). About 80% of the invited participants (*n* = 2,798) had scores ≥90th percentile on these items, while the remainder (*n* = 654) were randomly selected from MoBa. Thirty-five percent agreed to participate, and 1,195 children (mean age 3.5 years) took part in a one-day clinical assessment, including a diagnostic interview with mothers. Fifteen mothers later withdrew from MoBa, leaving 1,180 enrolled children who were followed up at 8 years of age. This study includes responders for children with available parent scores at ages 3.5 and 8 years (*n* = 783). Differences between responders and non-responders at age 8 years were described previously (Overgaard et al., 2023). In short, the results indicated that responders were slightly, but significantly, better educated than non-responders (responders: mean = 14.94 years, *SD* = 2.21; non-responders: mean = 14.65 years, *SD* = 2.35; *t* = 2.03, *p* = .05). The sex distribution was similar in both groups, with boys comprising 53% of responders and 52% of non-responders. Additionally, there were no significant differences in the number of parent-reported ADHD symptoms at age 3 years between responders and non-responders (responders: mean = 3.97, *SD* = 3.84; non-responders: mean = 4.21, *SD* = 3.99; *p* = .32). Furthermore, the number of teacher-reported Early Childhood Inventory-4 (ECI-4) ADHD symptoms was slightly, but significantly higher among responders (mean 2.50 (*SD* = 4.11)) than non-responders (mean 1.62 (*SD* = 3.05); *p* = .01).

## Measures

Child sex was obtained from the National Medical Birth Registry. Length of parental education was obtained at the first MoBa assessment with questionnaires to mothers (about Week 17 of pregnancy).

At 3.5 years of age, the semi-structured Preschool Age Psychiatric Assessment (PAPA) interview, developed for children aged 2 to 5 years (Egger & Angold, 2004), was conducted by trained interviewers, primarily with mothers. In the present study, we utilized symptom sum scores from the PAPA chapters for ADHD (hyperactivity-impulsivity (HI) and inattention (IA)), anxiety, and the behavioral disorder oppositional defiant disorder (ODD), considering only symptoms persisting for at least three months. A second blind rater independently rescored audiotapes of 79 randomly selected interviews, resulting in intraclass correlations of .97 and .99 for HI and IA, .74 for anxiety, .98 for ODD symptoms, and .94 for the total impairment score.

For ADHD, we computed mean scores from the nine respective symptoms of HI or IA subscales defined by the DSM-IV-TR (American Psychiatric Association [APA], 2000).

For anxiety disorders, we calculated mean scores from three symptoms of social phobia (Social anxiety, Fear of activities in public, Fear of social situations with adults), six symptoms of separation anxiety (Avoids being alone, Concern for separation, Anxious when caregiver is absent, Nightmares about separation, Anxiety about going to preschool, Apprehension of injury), and four symptoms of generalized anxiety (Worries overall, Problems of concentration when a caring person is absent, Easily tired when anxious/concerned, Unnecessary need for reassurance).

For ODD, we calculated mean scores for eight symptoms (Defiance, Argues with adults, Outburst of anger, Annoys on purpose, Blames others, Vengeful, Overly sensitive, Angry and irritable). All these scales demonstrated acceptable internal consistency (Cronbach’s alphas): HI/IA .91/.90, Social phobia .77, Separation anxiety .57, General anxiety .66, and ODD .56.

Teachers reported ADHD symptoms at age 3.5 years by the ECI-4 which contains nine items each for HI and IA, rated on a 4-point Likert scale (0 = never, 1 = sometimes, 2 = often, and 3 = very often) corresponding to the ADHD symptom lists in the DSM–IV (Gadow & Sprafkin, 2000) with acceptable psychometric properties (Overgaard et al., 2019). Approximately 95% of 3.5-year-oldchildren in Norway attended preschool during the period of inclusion.

At child age 8 years, parents completed the Child Symptom Inventory-4 (CSI-4), a questionnaire featuring items and algorithms for diagnoses derived from the DSM-IV diagnostic criteria (APA, 2000). Consistent with the manual, the CSI-4 was rated on a four-point Likert scale (never, sometimes, often, very often; range 0–3), providing sum severity scores (Gadow & Sprafkin, 2000). For HI and IA, the CSI-4 includes nine items for each subscale. Regarding social anxiety, three items were assessed (Avoids contact with strangers, Excessively shy with peers, Cries/freezes/withdraws when put in uncomfortable social situations). For separation anxiety, eight items were examined (Upset when expected to separate from home/parents, Worries that parents will be hurt or leave, Worries that some disaster will lead to separation, Tries to avoid going to school, Worries about being left at home alone or with a sitter, Afraid to go to sleep unless near parents, Nightmares about being separated from parents, Complains about feeling sick when expecting to be separated from home/parents). Generalized anxiety was evaluated with five items (Difficulty controlling worries, Restless, Irritable most of the day, Extremely tense/Unable to relax, Difficulty falling asleep/staying asleep), and ODD was assessed with eight items (Loses temper, Argues with adults, Defies/refuses, Annoys deliberately, Blames others, Touchy/easily annoyed, Angry/resentful, Takes out anger on others). All these scales demonstrated high internal consistency: HI/IA .88/.88, Social phobia .71, Separation anxiety .80, General anxiety .77, and ODD .89.

Teachers also completed the CSI-4 HI and IA subscales, each with the nine items described.

## Child Impairment at Ages 3.5- and 8-Year

The PAPA ADHD section and the CSI-4 questionnaire both concluded with six items assessing the functional impairment of the child in everyday life. Responses were recorded on a four-point Likert scale (no, a little, some, a great deal; range 0–3) across six domains: (1) family relationships; (2) child’s quality of life (QoL); (3) learning; (4) play/leisure activities; (5) friends; and (6) family burden. At both ages, high intercorrelations were observed between the family domains (1 and 6; *r* = .66 and .72), which were therefore collapsed into one domain: family relationships/burden, resulting in five impairment domains.

Teachers assessed child impairment in four functional domains: (1) child QoL, (2) impairment with friends, (3) learning, and (4) in the class at age 3.5 years (*n* = 316) and 8 years (*n* = 654).

Table 1 displays the sample’s parent mean symptom levels at ages 3.5 and 8 years (*n* = 783), revealing an increase in IA and the three anxiety dimension scores (*p* < .001), and a slight decrease in HI (*p* = .04). The same table displays the IA and HI data for teachers, indicating a stable level of IA and a clear drop in HI. In a previous study, we reported that for ADHD mean symptom scores, the correlations between parents and teachers were low at 3.5 years (*r* = .36) and higher at 8 years (*r* = .56; Overgaard et al., 2023). Note that while parent ratings were typically provided by the child’s mother at ages 3.5 and 8 years, teacher ratings were given by the preschool teacher at age 3.5 years and by the primary school teacher at age 8 years.

**Table 1.** Mean Parent- and Teacher-reported Symptom Scores at Ages 3.5 and 8 years.

Parent-reported symptoms	3.5 years			8 years			Change		
	*n*	Mean	*SD*	*N*	Mean	*SD*	*N*	Mean	*SD*
Hyperactivity-impulsivity	783	0.71	0.71	783	0.66	0.62	783	−0.05**	0.67
Inattention	783	0.33	0.47	783	0.83	0.59	783	0.50*	0.60
Oppositional defiant disorder	782	0.80	0.44	783	0.81	0.54	782	0.01	0.58
Social anxiety	783	0.07	0.33	777	0.27	0.42	777	0.21*	0.45
Separation anxiety	783	0.12	0.28	777	0.21	0.32	777	0.09*	0.36
Generalized anxiety	781	0.05	0.23	783	0.41	0.47	781	0.37*	0.51
Teacher-reported symptoms									
Hyperactivity-impulsivity	396	0.59	0.54	674	0.37	0.59	331	−0.20*	0.68
Inattention	396	0.59	0.47	675	0.55	0.63	331	−0.04	0.67

*Note*. At age 3.5 years, parent and teacher symptom scores were calculated from the Preschool Age Psychiatric Assessment interview and Early childhood Inventory-4, respectively. At age 8 years, both symptom scores were calculated from the Child Symptom Inventory-4.

* *p* < .001. **p = .04.

## Ethics

MoBa and the initial data collection were based on a license from the Norwegian Data Protection Agency and approval from the Regional Committees for Medical and Health Research Ethics. The MoBa cohort is presently regulated by the Norwegian Health Registry Act. The current study was approved by the Regional Committees for Medical and Health Research Ethics (2017/1276).

## Analytic Plan

We computed means and standard deviations for both symptom and impairment scores, employing paired t-tests to assess the significance of the changes. Additionally, we calculated Pearson’s correlation coefficient (*r*) to determine correlations between symptom and impairment scores. Finally, multiple linear regression analyses were employed to estimate the percentage of variance explained by the symptom scores and to identify which specific symptom scores contributed significantly to the explained variance.

## Results

**Figure 1.**
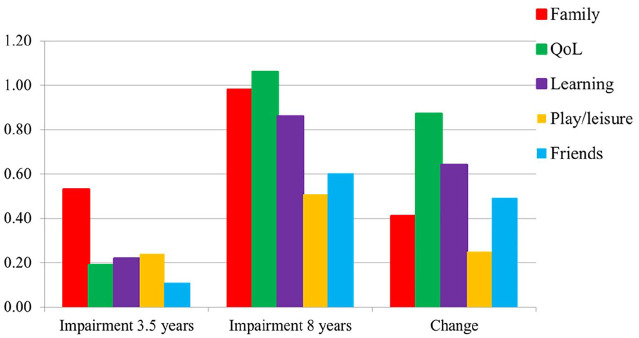
Parent-reported impairment scores for the five dimensions (family; child’s quality of life (QoL); learning; play/leisure activities; and friends) at ages 3.5 and 8 years and change over time.

**Figure 2.**
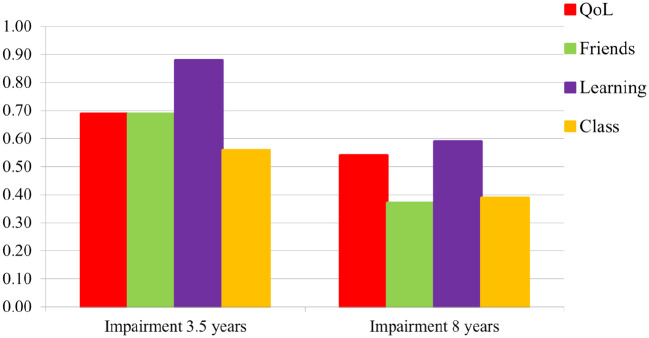
Teacher-reported impairment scores for the four dimensions (child quality of life (QoL), impairment with friends, learning, and in the class) at ages 3.5 and 8 years.

For parents, the mean global impairment score increased significantly from 0.30 (*SD* = 0.40) to 0.83 (*SD* = 0.63; *p* < .001) from 3.5 to 8 years. Figure 1 displays the mean parent item scores for the five different impairment dimensions (family; child’s QoL; learning; play/leisure activities; friends) at these time-points. All five impairment dimensions increased significantly (*p* < .001), with a particularly strong increase in impairment for QoL and learning. In contrast to parents, the teachers’ mean global impairment score slightly decreased from 0.70 (*SD* = 0.61) at 3.5 years to 0.47 (*SD* = 0.70) at 8 years. Figure 2 depicts the same four mean item scores at the two time-points for teachers, all showing the same trend.

As not all children fulfilled ADHD criteria at both time points, we checked whether the group who was not identified with ADHD in preschool but later at age 8 years differed from the children identified early. Between these groups, we did not find any significant differences in mean years of parent education 13.86 (*SD* = 2.19) versus 13.88 (*SD* = 2.22) or in the proportion of boys (65% vs. 64%). The teacher impairment scores for children identified with ADHD by parents at age 8 years were very similar to the same teacher scores for children identified with ADHD at ages 3.5 and 8 years. Specifically, the mean teacher impairment scores at ages 3.5 and 8 years, respectively, were as follows: 1.05 (*SD* = 0.66) and 1.30 (*SD* = 0.88) for the group with ADHD identified by parents at age 8 years only and 1.19 (*SD* = 0.75) and 1.30 (*SD* = 0.92) for the group identified with ADHD by parents at both time points.

We then assessed to what extent parent-reported preschool symptoms predicted the levels of impairment at 8 years. As shown in Table 2, the correlations with the 8-year impairment dimensions were strongest for HI, IA, and ODD, but overall, they were weak. In the multiple linear regression analyses, the age 3.5 symptom variables accounted for only 9% of the global impairment score variance. Among the specific impairment dimensions, the explained variance ranged from 4% (child’s QoL) to 8% (family; Supplemental Table 1).

**Table 2.** Correlations Between Parent-reported Child Symptoms at Age 3.5 Years and Impairment Scores at Age 8 Years (*n* = 533–561).

Impairment scores age 8 years	Child symptoms age 3.5 years					
	HI	IA	ODD	Social anxiety	Separation anxiety	Generalized anxiety
Global	.28	.25	.20	.00	.14	.03
Family	.21	.23	.23	-.01	.11	.01
Child QoL	.17	.13	.16	.02	.12	.03
Learning	.23	.16	.06	-.03	.13	.04
Play/leisure	.24	.21	.16	.07	.12	.05
Friends	.25	.24	.13	-.01	.08	.01

*Note.* At age 3.5 years, the symptom scores were calculated from the Preschool Age Psychiatric Assessment interview. For all coefficients above .10: *p* < .001. HI = hyperactivity-impulsivity; IA = inattention; ODD = oppositional defiant disorder; QoL = quality of life.

At age 8 years, the symptoms correlated moderately with impairment across all domains, particularly for HI, IA, ODD, and generalized anxiety symptoms (Table 3). At this age, the multiple linear regression analyses showed that the symptom variables accounted for 57% of the global impairment score variance, with all the different symptom scores (except separation anxiety) contributing significantly. For the different impairment dimensions, the contributions of explained variance from symptoms were similar to that of the global impairment score. For the learning impairment score, only IA and ODD symptoms contributed significantly, with IA explaining nearly all the variance (41%; Supplemental Table 2).

**Table 3.** Correlations Between Parent-reported Symptoms and Impairment at Age 8 years (*n* = 531–559).

Impairment scores age 8 years	Child symptoms age 8 years					
	HI	IA	ODD	Social anxiety	Separation anxiety	Generalized anxiety
Global	.60	.68	.59	.29	.32	.58
Family	.53	.56	.62	.27	.30	.57
Child /QoL	.41	.46	.50	.26	.33	.52
Learning	.44	.63	.25	.12	.16	.29
Play/leisure	.45	.51	.39	.25	.20	.42
Friends	.47	.48	.45	.23	.22	.43

*Note.* At age 8 years, the symptom scores were calculated from the Child Symptom Inventory-4. For all coefficients: *p* < .001. HI = hyperactivity-impulsivity; IA = inattention; ODD = oppositional defiant disorder; QoL = quality of life.

For teachers, the preschool IA- and HI-symptom scores were only weakly related to the teacher-reported 8-year global impairment score (IA = 0.31, HI = 0.30), whereas the 8-year symptom scores were significantly correlated with the global impairment score (IA = 0.81, HI = 0.76). At 8 years the symptom scores were also strongly correlated with the specific impairment dimensions, particularly IA with Learning (*r* = .84).

While teachers reported a lower level of impairment at 8 years than parents did, the global impairment scores between informants were moderately correlated (*r* = .55), and the correlation was even stronger for learning (*r* = .64; Table 4).

**Table 4.** Correlations Between Parent and Teacher-reported Impairment Ratings at 8 Years (*n* = 440–468).

	Teacher ratings				
	Global	Child QoL	Friends	Learning	Class
Parent ratings					
Impairment	.55	.51	.49	.50	.43
Family	.37	.34	.34	.32	.30
Child QoL	.32	.33	.31	.25	.25
Learning	.61	.58	.44	.64	.49
Play/leisure	.45	.43	.41	.40	.36
Friends	.43	.38	.46	.37	.32

*Note.* QoL = quality of life. For all coefficients *p* < .001.

**Figure 3.**
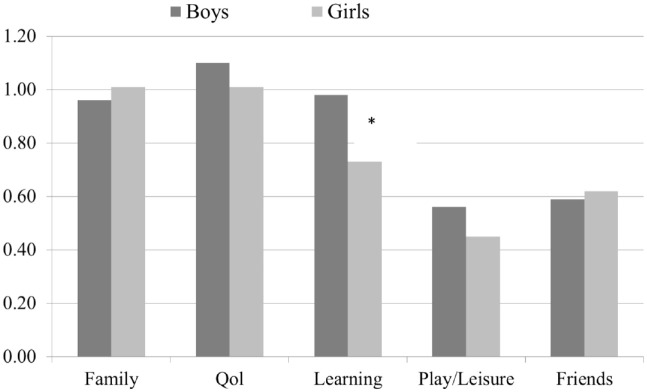
Parent-reported impairment dimension scores for boys and girls at age 8 years. *p < .001

**Figure 4.**
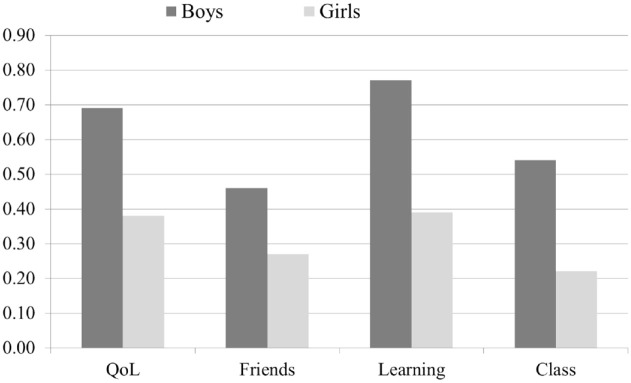
Teacher-reported impairment dimension scores for boys and girls at age 8 years. *p* < .001 for all dimensions.

Figure 3 shows that there were only minor sex differences concerning parent impairment scores, with one important exception. Boys at age 8 years had a significantly higher parent impairment score within the learning dimension (*p* < .001). Teachers reported significantly higher levels of global impairment for boys than for girls both in preschool and at 8 years. However, at 3.5 years, the difference was moderate (boys: mean = 0.77 (*SD* = 0.67), girls: mean = 0.59 (*SD* = 0.48), *p* < .02), while at 8 years, the mean score for boys was almost double that of girls (boys: mean = 0.61 (*SD* = 0.77), girls: mean = 0.32 (*SD* = 0.56) *p* < .001). In contrast to parents, teachers rated boys at 8 years with significantly (*p* < .001) higher impairment scores than girls for all dimensions (Figure 4).

## Discussion

This study unveiled several key insights into functional impairment related to ADHD and its comorbidities from 3.5 to 8 years. In accordance with our first hypothesis, parent-reported impairment levels increased globally and for all dimensions during this period. This rise was particularly pronounced for dimensions related to quality of life and learning. Two longitudinal studies, commencing after the age of 4 years, found stable overall impairment extending into school age (Lahey et al., 2016; Leopold et al., 2019). This disparity between these studies and ours, indicates that child impairment may be less discernible in young preschoolers. Furthermore, a study with the initial assessment conducted during early childhood (4–6 years) revealed that despite improvements in ADHD symptoms during adolescence, impairment persisted (Lee et al., 2008). Again, this observation is likely influenced by the study’s recruitment of older children compared to our study. Additionally, supporting the significance of age at inclusion, a comparative study examining two cohorts of children with ADHD noted substantial global impairment in both the preschool group (ages 4–6 years; *n* = 165) and the school-age group (ages 7–9 years; *n* = 381), as well as impairments in school and socially (Wilens et al., 2002). However, more in line with our findings, a recent study comparing children over and below the age of 11 years found that older children were significantly more impaired even though they had fewer symptoms (De Rossi et al., 2023). Contrary to our first hypothesis though, we observed a slight decrease in teacher-reported mean impairment scores from preschool to school age. This finding may seem surprising, given the increasing academic demands typically associated with age. However, several factors could have contributed to this result. One factor is that teachers gathered information from distinctly different settings: preschool and school. Also, we only had teacher reports for a smaller sub-sample. Furthermore, unlike parental scores, the teacher assessments were conducted by different individuals at the two time points. Additionally, a study revealed gaps in preschool teachers’ knowledge of ADHD, reporting only 38.3% accuracy, with the greatest inaccuracies related to ADHD symptoms and treatment (Poznanski et al., 2020). Finally, a study (*n* = 2,140; age 5–17 years) reported that teacher ratings of ADHD symptoms and impairments (for all domains) decreased with child age (DuPaul et al., 2014). Together, these studies suggest that clinicians should be cautious when interpreting teacher impairment scores, at least for preschool children with ADHD.

In line with our second hypothesis, we found that parent-reported preschool symptom scores significantly, but only weakly, predicted impairment scores at 8 years. This is consistent with another population study with the first assessment at age 3.6 years (Finsaas et al., 2020). However, this weak association contrasted somewhat with a clinical study (*n* = 255, 82% males) reporting that ADHD at age 4 to 6 years moderately predicted poorer social and academic functioning in adolescence (Lee et al., 2008). A population-based twin study beginning in preschool found that parent-reported HI and IA symptoms increased the risk of concurrent and future global, social, and recreational impairment, while IA was significantly associated with academic impairment (Leopold et al., 2019). The stronger associations found in these studies compared to our findings may be attributed to their participants being older preschoolers at their first assessments than ours. This assumption is supported by the fact that we too found a clear shift toward stronger correlations between symptom scores and impairment at age 8 years compared to at 3.5 years. Particularly, IA, ODD, and general anxiety symptoms showed robust associations with impairment at age 8 years. This aligns with our third hypothesis and with a European study linking poor global impairment with ADHD symptom severity, oppositional defiance, and anxiety symptoms (Coghill et al., 2006). Additionally, a clinical study reported greater homework problems when children with ADHD had comorbid disorders compared to those without such disorders (Booster et al., 2012). Scrutinizing the impairment dimensions at 8 years, IA symptoms explained most of the variance of the learning impairment score, in line with the robust literature described in a review (O’Neill et al., 2017). Although we found significant correlations between parent and teacher impairment scores at both ages, it was weak at 3.5 years, indicating low agreement across settings. At age 8 years, the parent-teacher agreement had increased considerably.

In line with our fourth hypothesis, we found only minor sex differences in parent impairment scores, resembling the aforementioned European study reporting similar patterns of parent-reported impairment in boys and girls, although the domains of impairment in that study differed from ours (e.g., bullying, truancy, visits to emergency facilities, and contact with police/child social services; Novik et al., 2006). However, in our study, we did reveal one important exception for parents: at age 8 years, boys were significantly more impaired within the learning dimension. This may be in accordance with a study of school-age children where boys with ADHD had lower reading performance (reading task) than girls at grade levels 1 to 4 (*n* = 2,014; Ehm et al., 2016), but is contrary to findings of lower academic achievements in girls with ADHD compared to boys (*n* = 998, age 11 years; Elkins et al., 2011). The disparity between these studies, may partly be explained by the participant’s age. Also, the 2022 Annual research review on ADHD in girls, where the authors reported contradictory outcomes regarding whether girls with ADHD have equivalent or greater academic achievements as boys and point to both referral source and informant as crucial moderators here (Hinshaw et al., 2022).

Finally, in line with our fifth hypothesis, teachers consistently reported significantly higher levels of global impairment for boys compared to girls at both 3.5 and 8 years, aligning with a prior study indicating elevated teacher-rated impairment levels in preschool boys compared to girls (Healey et al., 2008). In contrast to parents, teachers rated the 8-year-old boys as significantly more impaired than girls for all dimensions. This seems in accordance with a large study showing that teachers reported lower levels of ADHD symptoms in girls than in boys (Overgaard et al., 2022). This pattern raises concerns that girls with ADHD might be overlooked by teachers, potentially due to their quieter demeanor in class. If girls present with minimal disruptions in the preschool or school setting, teachers may be at risk of not noticing impairment in other areas, such as quality of life and relationships with friends. A study with only girls showed that those with childhood ADHD demonstrated lower rates of social skills, social preference, and academic achievement during adolescence compared to their non-ADHD counterparts. In fact, only 40.5 % of the girls with ADHD demonstrated adequate parent-reported social skills during adolescence compared to 82.7% of the comparisons (Owens et al., 2009). Also, using teacher-reports, that same study reported a significant but smaller sex difference than parents, with 61.1% of the girls with childhood ADHD receiving adequate social preference ratings compared to 87.7% of the comparisons, suggesting that teachers might find it easier to observe impairment in adolescent girls than they do at age 8 years.

## Strengths and Limitations

The strengths of the present study were the relatively large sample and the first assessment in early preschool, as well as having both global impairment and dimension scores from both parents and teachers. Several limitations must be emphasized. Selection bias due to attrition has been well-documented (Magnus et al., 2016; Overgaard et al., 2019). The minimal differences between responders and non-responders at the 8-year follow-up suggest that attrition is unlikely to have significantly biased the results.

Unfortunately, the teacher data at age 3.5 years were diminished by using two different questionnaires for ADHD, only having available ECI-4 on about half the sample. With this limitation and the known low symptom agreement between parents and teachers at age 3.5 years, as previously shown (Overgaard et al., 2022, 2023), only teacher-reported HI and IA symptoms by ECI-4 were included in the present study. We also included teacher impairment data from this subsample. The higher teacher impairment scores at age 3.5 years compared to parents may be because the teachers responded to a questionnaire with general questions about impairment, while parents were specifically interviewed about impairment when they reported ADHD symptoms as present. However, a previous study pointed out that parent ratings at age 3.5 years likely reflected global impairment, as the impairment scores were strongly correlated between across the PAPA chapters (for ADHD and ODD (*r* = .65); for ADHD and Conduct disorder (*r* = .90); Bendiksen et al., 2014). The slight decrease in teacher impairment scores across age, might result from a slight change in wording of the items from preschool to school. Generally, the lack of unified measures for impairment dimensions makes it difficult to compare between studies. The fact that the teachers were entirely different individuals at 3.5 and 8 years adds to the uncertainty.

## Conclusions and Future Directions

The present study contributes to existing literature by demonstrating that parents consistently report a significant and wide-ranging increase across impairment dimensions from preschool to school age. Overall, we propose that early preschool ADHD symptoms may have limited predictive power for impairment extending into school age. The associations between HI, IA, comorbid anxiety, and behavioral symptoms and reported impairment notably strengthened from preschool to school age. While the magnitude of contribution varied among different dimensions of impairment, the overarching trend suggests that all these symptoms play a role. For teachers, a noteworthy finding was the robust correlation observed between IA symptoms and learning impairment, alongside a substantially higher impairment score reported for boys compared to girls. Future studies should highlight the importance of understanding the dynamics between ADHD and comorbid symptoms and impairment over time and across settings, with a specific focus on developing measures for teachers to identify impairment in schoolgirls.

## Supplemental Material

sj-docx-1-jad-10.1177_10870547241301179 – Supplemental material for Functional Impairment Related to ADHD From Preschool to School Age Supplemental material, sj-docx-1-jad-10.1177_10870547241301179 for Functional Impairment Related to ADHD From Preschool to School Age by Kristin Romvig Overgaard, Beate Oerbeck, Svein Friis, Are Hugo Pripp, Heidi Aase, Christine Baalsrud Ingeborgrud and Guido Biele in Journal of Attention Disorders

sj-docx-2-jad-10.1177_10870547241301179 – Supplemental material for Functional Impairment Related to ADHD From Preschool to School Age Supplemental material, sj-docx-2-jad-10.1177_10870547241301179 for Functional Impairment Related to ADHD From Preschool to School Age by Kristin Romvig Overgaard, Beate Oerbeck, Svein Friis, Are Hugo Pripp, Heidi Aase, Christine Baalsrud Ingeborgrud and Guido Biele in Journal of Attention Disorders
