# Supplementary material for: Functional Impairment Related to ADHD From Preschool to School Age
Source: J Atten Disord. 2024 Dec 3;29(3):220–30. doi: 10.1177/10870547241301179 (PMC11694549; doi:10.1177/10870547241301179)
Supplement: sj-docx-1-jad-10.1177_10870547241301179 – Supplemental material for Functional Impairment Related to ADHD From Preschool to School Age [file sj-docx-1-jad-10.1177_10870547241301179.docx]

**Table S1** *Multiple linear regression analyses -Parent-reported 3-years symptom variables with significant future significant contributions to explained variance of impairment at age 8 years*

|  | **Impairment age 8 years** | | | | | | | | | | | | | | | | | | | | |
| --- | --- | --- | --- | --- | --- | --- | --- | --- | --- | --- | --- | --- | --- | --- | --- | --- | --- | --- | --- | --- | --- |
|  | Global |  |  |  |  |  |  | Family |  |  |  |  |  |  | Child /QoL |  |  |  |  |  |  |
| **Mean symptom scores, age 3 years** | t | *p* | β | F | df | *p* | R² | t | *p* | β | F | df | *p* | R² | t | *p* | β | F | df | *p* | R² |
| Overall model |  |  |  | 20.2 | 3 | <.001 | .09 |  |  |  | 23.5 | 2 | <.001 | .08 |  |  |  | 11.3 | 2 | <.001 | .04 |
| HI | 3.27 | <.001 | .11 |  |  |  |  |  |  |  |  |  |  |  | 2.76 | .006 | .12 |  |  |  |  |
| IA | 2.07 | .039 | .11 |  |  |  |  | 4.06 | <.001 | .17 |  |  |  |  |  |  |  |  |  |  |  |
| ODD | 2.66 | .008 | .17 |  |  |  |  | 4.05 | <.001 | .17 |  |  |  |  | 2.82 | .005 | .13 |  |  |  |  |
| Social Anxiety |  |  |  |  |  |  |  |  |  |  |  |  |  |  |  |  |  |  |  |  |  |
| Separation Anxiety |  |  |  |  |  |  |  |  |  |  |  |  |  |  |  |  |  |  |  |  |  |
| GAD |  |  |  |  |  |  |  |  |  |  |  |  |  |  |  |  |  |  |  |  |  |

|  | **Impairment age 8 years** | | | | | | | | | | | | | | | | | | | | |
| --- | --- | --- | --- | --- | --- | --- | --- | --- | --- | --- | --- | --- | --- | --- | --- | --- | --- | --- | --- | --- | --- |
|  | Learning |  |  |  |  |  |  | Play/  Leisure |  |  |  |  |  |  | Friends |  |  |  |  |  |  |
| **Mean symptom scores, age 3 years** | t | *p* | β | F | df | *p* | R² | t | *p* | β | F | df | *p* | R² | t | *p* | β | F | df | *p* | R² |
| Overall model |  |  |  | 17.5 | 2 | <.001 | .06 |  |  |  | 18.4 | 2 | <.001 | .06 |  |  |  | 20.7 | 2 | <.001 | .07 |
| HI | 4.97 | <.001 | .21 |  |  | <.001 |  | 4.77 | <.001 | .21 |  |  |  |  | 2.98 | .003 | .16 |  |  |  |  |
| IA |  |  |  |  |  |  |  |  |  | . |  |  |  |  | 2.55 | .011 | .14 |  |  |  |  |
| ODD |  |  |  |  |  |  |  | 1.99 | .047 | .17 |  |  |  |  |  |  |  |  |  |  |  |
| Social Anxiety |  |  |  |  |  |  |  |  |  |  |  |  |  |  |  |  |  |  |  |  |  |
| Separation Anxiety | 2.11 | .035 | .89 |  |  | .035 |  |  |  |  |  |  |  |  |  |  |  |  |  |  |  |
| GAD |  |  |  |  |  |  |  |  |  |  |  |  |  |  |  |  |  |  |  |  |  |

HI hyperactivity-impulsivity; IA inattention; ODD oppositional defiant disorder; GAD Generalized anxiety disorder. Only variables with significant contributions were included in the models.
