# Supplementary material for: Functional Impairment Related to ADHD From Preschool to School Age
Source: J Atten Disord. 2024 Dec 3;29(3):220–30. doi: 10.1177/10870547241301179 (PMC11694549; doi:10.1177/10870547241301179)
Supplement: sj-docx-2-jad-10.1177_10870547241301179 – Supplemental material for Functional Impairment Related to ADHD From Preschool to School Age [file sj-docx-2-jad-10.1177_10870547241301179.docx]

**Table S2** *Multiple linear regression analyses* **-** *Parent-reported 8-years symptom variables with significant concurrent contributions to explained variance of impairment at age 8 years*

|  | **Impairment age 8 years** | | | | | | | | | | | | | | | | | | | | |
| --- | --- | --- | --- | --- | --- | --- | --- | --- | --- | --- | --- | --- | --- | --- | --- | --- | --- | --- | --- | --- | --- |
|  | Global |  |  |  |  |  |  | Family |  |  |  |  |  |  | Child /QoL |  |  |  |  |  |  |
| **Means symptom scores, age 8 years** | t | *p* | β | F | df | *p* | R² | t | *p* | β | F | df | *p* | R² | t | *p* | β | F | df | *p* | R² |
| Overall model |  |  |  | 145.6 | 5 | <.001 | .57 |  |  |  | 133.2 | 4 | <.001 | .49 |  |  |  | 71.0 | 4 | <.001 | .35 |
| HI | 3.43 | <.001 | .14 |  |  |  |  | 2.33 | .02 | .10 |  |  |  |  |  |  |  |  |  |  |  |
| IA | 9.46 | <.001 | .38 |  |  |  |  | 5.19 | <.001 | .22 |  |  |  |  | 4.62 | <.001 | .20 |  |  |  |  |
| ODD | 5.22 | <.001 | .20 |  |  |  |  | 8.45 | <.001 | .35 |  |  |  |  | 4.79 | <.001 | .23 |  |  |  |  |
| Social Anxiety | 2.98 | .003 | .09 |  |  |  |  |  |  |  |  |  |  |  |  |  |  |  |  |  |  |
| Separation Anxiety |  |  |  |  |  |  |  |  |  |  |  |  |  |  | 2.90 | .004 | .12 |  |  |  |  |
| GAD | 3.58 | <.001 | .15 |  |  |  |  | 4.00 | <.001 | .17 |  |  |  |  | 3.91 | <.001 | .21 |  |  |  |  |

|  | **Impairment age 8 years** | | | | | | | | | | | | | | | | | | | | |
| --- | --- | --- | --- | --- | --- | --- | --- | --- | --- | --- | --- | --- | --- | --- | --- | --- | --- | --- | --- | --- | --- |
|  | Learning |  |  |  |  |  |  | Play/  Leisure |  |  |  |  |  |  | Friends |  |  |  |  |  |  |
| **Mean symptom scores, age 8 years** | t | *p* | β | F | df | *p* | R² | t | *p* | β | F | df | *p* | R² | t | *p* | β | F | df | *p* | R² |
| Overall model |  |  |  | 90.9 | 2 | <.001 | .41 |  |  |  | 24,1 | 4 | <.001 | .31 |  |  |  | 59.9 | 4 | <.001 | .31 |
| HI |  |  |  |  |  |  |  | 2.92 | .004 | .16 |  |  |  |  | 4.21 | <.001 | .22 |  |  |  |  |
| IA | 17.8 | <.001 | .38 |  |  |  |  | 6.09 | <.001 | .31 |  |  |  |  | 3.99 | <.001 | .20 |  |  |  |  |
| ODD | -2.58 | .01 | -.01 |  |  |  |  |  |  |  |  |  |  |  | 4.28 | <.001 | .19 |  |  |  |  |
| Social Anxiety |  |  |  |  |  |  |  | 3.34 | .002 | .13 |  |  |  |  | 2.91 | .004 | .11 |  |  |  |  |
| Separation Anxiety |  |  |  |  |  |  |  |  |  |  |  |  |  |  |  |  |  |  |  |  |  |
| GAD |  |  |  |  |  |  |  | 2.56 | .011 | .12 |  |  |  |  |  |  |  |  |  |  |  |

HI hyperactivity-impulsivity; IA inattention; ODD oppositional defiant disorder; GAD Generalized anxiety disorder. Only variables with significant contributions were included in the models.
